# Regulation of Eukaryotic Initiation Factor 4AII by MyoD during Murine Myogenic Cell Differentiation

**DOI:** 10.1371/journal.pone.0087237

**Published:** 2014-01-23

**Authors:** Gabriela Galicia-Vázquez, Sergio Di Marco, Xian J. Lian, Jennifer F. Ma, Imed E. Gallouzi, Jerry Pelletier

**Affiliations:** 1 Department of Biochemistry, McGill University, Montreal, Quebec, Canada; 2 The Rosalind and Morris Goodman Cancer Research Center, McGill University, Montreal, Quebec, Canada; The John Curtin School of Medical Research, Australia

## Abstract

Gene expression during muscle cell differentiation is tightly regulated at multiple levels, including translation initiation. The PI3K/mTOR signalling pathway exerts control over protein synthesis by regulating assembly of eukaryotic initiation factor (eIF) 4F, a heterotrimeric complex that stimulates recruitment of ribosomes to mRNA templates. One of the subunits of eIF4F, eIF4A, supplies essential helicase function during this phase of translation. The presence of two cellular eIF4A isoforms, eIF4AI and eIF4AII, has long thought to impart equivalent functions to eIF4F. However, recent experiments have alluded to distinct activities between them. Herein, we characterize distinct regulatory mechanisms between the eIF4A isoforms during muscle cell differentiation. We find that eIF4AI levels decrease during differentiation whereas eIF4AII levels increase during myofiber formation in a MyoD-dependent manner. This study characterizes a previously undefined mechanism for eIF4AII regulation in differentiation and highlights functional differences between eIF4AI and eIF4AII. Finally, RNAi-mediated alterations in eIF4AI and eIF4AII levels indicate that the myogenic process can tolerate short term reductions in eIF4AI or eIF4AII levels, but not both.

## Introduction

Assembly of eukaryotic Initiation Factor (eIF) 4F complex is rate-limiting for protein synthesis and is required for efficient ribosome recruitment to mRNA templates. The eIF4F complex is composed of three proteins, namely eIF4E, a cap binding protein; eIF4A, an ATP dependent RNA helicase; and eIF4G, a scaffolding protein that bridges interactions between the mRNA and ribosome-bound eIF3 [1]. EIF4A is an abundant factor that is present as a free form (eIF4A_f_) or as a part of the eIF4F complex (eIF4A_c_). The helicase activity of eIF4A_c_ is ∼20 fold more active than that of eIF4A_f_, suggesting that eIF4A_c_ is mainly responsible for helicase activity during initiation [2]. There are two isoforms of eIF4A, eIF4AI and eIF4AII, which are 90% identical at the amino acid level and functionally interchangeable *in vitro* [3]–[5]. It has also been documented that eIF4AI and eIF4AII are differentially expressed and their ratios differ in various tissues [3], [6]. However, *in vivo* eIF4AII cannot compensate for the suppression of eIF4AI, indicating different roles for the two isoforms [7]. As well, eIF4AII, but not eIF4AI, has been implicated in miRNA repression of mRNA expression [8]. Previously, as a result of a screen for translation inhibitors, we identified three compounds, hippuristanol, silvestrol, and pateamine A, which curtail cap dependent translation by targeting eIF4AI and eIF4AII [9]–[14].

C2C12 cells have been valuable for establishing some of the general principles for myogenic differentiation. In this model, MyoD is activated leading to induction of p21 and myogenin - essential drivers of the myogenic process that promote cell cycle arrest and cell fusion, respectively [15], [16]. An additional layer of influence on the myogenic process is signalling by the PI3K/mTOR pathway and its role in regulating translation. Specifically, protein synthesis rates increase within the first 24 h of C2C12 differentiation and this correlates with an increase in phosphorylation of the eIF4E repressor, 4EBP1, as well as phosphorylation of eIF4E at serine 209 – two events that have been linked to increased translation initiation rates [17]. Moreover, rapamycin or RAD001, inhibitors of mTOR signalling and cap-dependent translation, block muscle cell differentiation [17]–[19]. Herein we document differential expression of the eIF4A isoforms during muscle differentiation and report that part of this response is MyoD-dependent.

## Materials and Methods

### Cell culture

C2C12 myoblasts (ATCC) were grown in DMEM (Invitrogen) supplemented with 20% FBS and 100 U/ml penicillin/streptomycin at 37°C and 5% CO_2_. To induce differentiation, cells were grown to confluency, at which point the culture media was changed to media containing DMEM, 2% horse serum, and 100 U/ml penicillin/streptomycin (referred to as differentiation media or DM). Primary myoblasts were maintained in DMEM supplemented with 20% FBS, 10% horse serum, 1% chicken embryo extract, 100 U/ml penicillin/streptomycin and 0.2% fungizone (Gibco). To induce differentiation, the culture media was changed to DMEM supplemented with 2% FBS, 10% horse serum, 0.5% chicken embryo extract, 100 U/ml penicillin/streptomycin and 0.2% fungizone. NIH-3T3 cells were cultured in DMEM supplemented with 10% FBS, and 100 U/ml penicillin/streptomycin at 37°C and 5% CO_2_.

### Immunoblot Analysis and ^35^S-Met/Cys labeling

Protein samples were fractionated on SDS-polyacrylamide gels, and transferred to PVDF membranes (Bio-Rad). Antibodies used in this study were directed against: eIF4AI (ab31217; Abcam), eIF4AII (ab31218; Abcam), eIF4E (sc9976; Santa Cruz Biotech), eIF4GI (A300-502A; Bethyl Labs), PDCD4 (9535; Cell Signaling Tech), myogenin (F5D; Developmental Studies Hybridoma Bank), MyoD (M-318, sc-760, Santa Cruz Biotech), GAPDH (ab8245; Abcam), and β-actin (A5441; Sigma). ^35^S-methionine/cysteine protein labeling was performed as described previously [7].

### Reverse Transcription and Quantitative PCR

Cellular RNA was isolated using TRIzol (Invitrogen). cDNA was generated by reverse transcription using SuperScript III and oligo d(T)_(12–18)_ primers according to the manufacturer's instructions (Invitrogen). Quantitative PCRs were set up using SsoFast Evagreen Supermix (Bio-Rad) and performed in a CFX96 PCR System (Bio-Rad). The data was analyzed using Bio-Rad CFX Manager 2.1 software. Threshold cycles (C_T_) were determined by single threshold and the relative amounts of eIF4AI, eIF4AII, and eIF4E mRNA elucidated by the ΔΔC_T_ method. Primer efficiencies were determined and taken into account in the C_T_ expression determinations. Primers targeting eIF4AI, eIF4AII, MyoD, and GAPDH have been previously described [7], [20]. Primers used to detect eIF4E and myogenin were: eIF4E-For (^5′^TGCCTGGCTGTGACTACTCACTTT^3′^), eIF4E-Rev (^5′^GTCTCTGCTGTTTGTTCAATGTAA^3′^), MyoG-For (^5′^CTACAGGCCTTGCTCAGCTC^3′^), and MyoG-Rev (^5′^AGATTGTGGGCGTCTGTACG^3′^).

### m^7^GTP Sepharose Pull-Down Assays

C2C12 cells were grown in 150 cm^2^ dishes and induced for differentiation. During the differentiation process, cells were harvested, extracts prepared, and pull-down experiments performed as described [7].

### Nuclear Run On

Nuclear run-ons were performed as described [21]. Probes for eIF4AII at 5′UTR ([NM_013506] positions 1 to 329), eIF4AII at 3′UTR ([NM_013506] positions 1570 to 2059), MyoD ([NM_010866] positions 1 to 510) and GAPDH ([NM_008084] positions 1 to 499) were prepared and used in this assay.

### RNAi-mediated Suppression

For knockdown experiments, C2C12 cells were transfected with siRNAs twice. The first transfection was performed when cells were 25–30% confluent. Twenty four hours later, cells were transfected a second time (when they had reached 50–60% confluency). Differentiation was induced 24 hrs after the second transfection and cells were harvested at the indicated time points. Transfections were performed using JetPrime reagent according to the manufacturer's recommendations (Polyplus). siRNAs used in this study were non-targeting (NT) siRNA (D-001206-13), siRNAs targeting mouse eIF4AI (M-060466-01), and mouse eIF4AII (M-042407-01) (siGENOME Smart Pool, Thermo Scientific).

### Chromatin Immunoprecipitation Assay

Putative MyoD binding sites were determined using TFSearch software [22]. C2C12 cells were grown in 150 cm^2^ dishes, induced to differentiate for 3 days, and samples were collected every 24 h. Chromatin was crosslinked by treating cells with 1% formaldehyde for 10 min. Cells were lysed in Lysis buffer (50 mM Tris_8.0_, 10 mM EDTA, 1% SDS, 20 mM β-glycerophosphate, 10 mM NaF, 1 mM PMSF, 4 mg/ml aprotinin, 2 mg/ml leupeptin, 2 mg/ml pepstatin) and crosslinked chromatin was sonicated to generate fragments of 500–1000 bp in length. The DNA was diluted with 1% Triton X-100, 2 mM EDTA, 20 mM Tris_8.0_, 150 mM NaCl and extracts were pre-cleared with salmon sperm DNA/Protein A-agarose (Upstate) and 300 µg BSA. Immunoprecipitations were performed with either anti-MyoD antibody (M-318, sc-760, Santa Cruz Biotech) or IgG control at 4°C overnight with tubes rotating end-over-end. The immunoprecipitates were washed once with Wash 1 (1% Triton X-100, 0.1% SDS, 2 mM EDTA, 20 mM Tris_8.0_, 150 mM NaCl), once with Wash 2 (1% Triton X-100, 0.1% SDS, 2 mM EDTA, 20 mM Tris_8.0_, 500 mM NaCl), and once with Wash 3 (0.25 M LiCl, 1% NP-40, 1% deoxycholate, 1 mM EDTA, 10 mM Tris_8.0_). A last wash was performed with TE buffer (10 mM Tris_8.0_, 1 mM EDTA) and the protein-DNA crosslinks were reversed by incubating in 1% SDS/0.1 M NaHCO_3_ at 65°C for 18 h. Samples were treated with RNase A and Proteinase K at 55°C for 1 h and DNA was purified using EZ-10 spin columns according to the manufacturer's instructions (Biobasic Inc). The presence of specific DNA sequences was detected by PCR using the following primers: eIF4AI-CHIPFor: (^5′^GGCCTCAAAATAGTGGCTGTGC^3′^), eIF4AI-CHIPRev: (^5′^GTATGTTTCCAGTTTCTCCTGGGC^3′^) positions: −667 to −483, eIF4AII-CHIPFor: (^5′^GTTACAAAGAATGACAGGTCCTTTGC^3′^), eIF4AII-CHIPRev: (^5′^TCATTAACAGATGTCCCTAGGGTGG^3′^) positions −545 to −377. Myogenin-CHIPFor: (^5′^GGAATCACATGTAATCCACTG^3′^) and Myogenin-CHIPRev: (^5′^TCACACCAACTGCTGGGT^3′^) positions −142 to −1 (positions are relative to the transcription start site (+1)).

### Promoter Activity Analysis

The mouse eIF4AI and eIF4AII proximal promoter sequences (positions −791 to −13 and −757 to +328) were amplified by PCR and cloned into phRL-null recipient vector (Promega). A vector containing mutations in the three MyoD binding sites in the eIF4AII promoter was generated by *de novo* synthesis (Genscript) and cloned into the BglII sites of phRL-4AII. The three MyoD binding site mutations introduced into the eIF4AII promoter were: (i)^−533^GACAGGTCCT^−524^ to ^−533^GAACTTGTCT^−524^, (ii) ^−487^CGCACCTGTT^−478^ to ^−487^CGCTAAGTTT^−478^, and (iii) ^−392^AACAGATGTC^−383^ to ^−392^AAACAAGTTC^−383^. The myogenin promoter cloned in pGL3 was a kind gift of Dr. M. Rudnicki (Ottawa Hospital Research Institute, Ottawa, Canada).

Transfections were performed in NIH-3T3 cells using JetPrime transfection reagent by adding 1 µg of phRL-4A reporter or pGL3-Myogenin control vector, and either 1 µg of pCS2 empty vector or pCS2-MyoD vector. Cell culture media was changed to differentiation media 24 h after transfection (Day 0) [23]. Extracts were prepared by lysing cells with Passive Lysis Buffer (Promega) at the indicated time points. Firefly and Renilla Luciferase activity (RLU) were quantitated with a Dual Luciferase Assay kit (Promega) on a Berthold Lumat LB 9507 luminometer.

### Immunofluorescence

Immunofluorescence was performed as previously described [20]. Cells were probed with antibodies against myosin heavy chain (MF-20, Developmental Studies Hybridoma Bank) and myoglobin (ab77232, Abcam). Cell differentiation was determined by calculating the fusion index of muscle fibers (the ratio of the number of nuclei in a microscope field per number of nuclei in myofibers). Myofiber diameter was measured using AxioVision Rel 4.8 software (Carl Zeiss). Ten fields from each independent experiment were analyzed.

### Data Analysis

Statistical analysis (Student's-t tests) was performed using GraphPad InStat version 3.10 (San Diego, CA).

## Results

### EIF4A isoforms are differentially expressed during muscle differentiation

**Figure 1:**
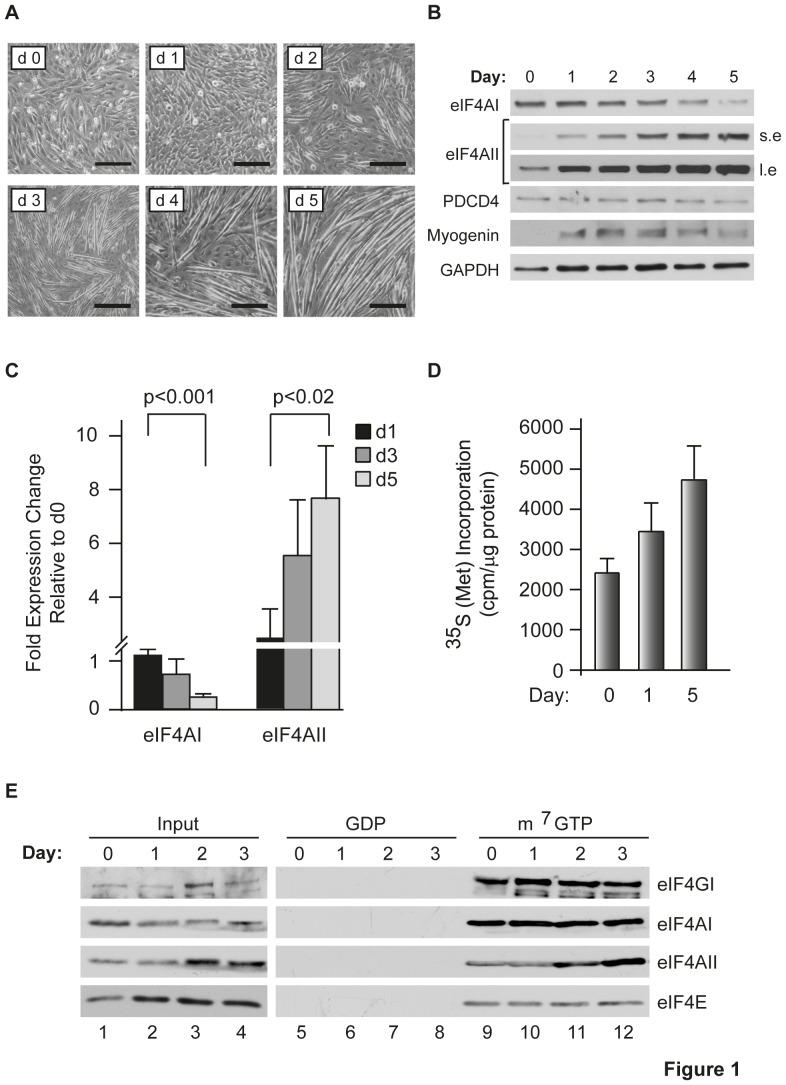
Expression of eIF4AI and eIF4AII during C2C12 differentiation. (**A**) Phase contrast images of C2C12 cells grown in the presence of DM for the indicated number of days (d). Scale bars represent 50 µm. (**B**) Western Blot analysis documenting expression levels of the indicated proteins during C2C12 cell differentiation. Long (l.e.) and short (s.e.) exposures of the eIF4AII Western blot are presented. (**C**) Quantification of changes in eIF4AI and eIF4AII protein levels relative to those obtained on day 0. n = 3±SEM. (**D**) ^35^S-methionine/cysteine incorporation into TCA-insoluble protein. C2C12 cells were induced to differentiate and protein extracts were prepared at the indicated time points. Cells were labeled for 30 min and the amount of radiolabeled protein quantitated by TCA precipitation. Values are standardized against total protein content. n = 3±SEM. (**E**) eIF4AI/II are efficiently incorporated into the eIF4F complex during C2C12 differentiation. m^7^GTP affinity purification of the eIF4F complex from C2C12 cells at the indicated days following induction of differentiation. Western blots to the indicated proteins were performed on an aliquot of input extract (lanes 1–4), GDP eluents (lanes 5–8), and m^7^GTP eluents (lanes 9–12).

We have previously reported that eIF4AI and eIF4AII do not appear to be equivalent in their contribution to cellular translational output [7]. In documenting differences in the ratio of eIF4AI and eIF4AII isoforms between fetal and adult tissues [7] and changes that occur as a consequence of differentiation, we found that during C2C12 myoblast differentiation there was a striking change in eIF4AI and eIF4AII protein levels (Figs. 1A, B). When exposed to DM, C2C12 cells fully differentiate into myoblasts by 3–4 days under our experimental conditions (Fig. 1A). Using isoform specific antibodies, we found that eIF4AI levels were fairly constant until d2–d3 of differentiation, at which point they began declining (Fig. 1B). In contrast, eIF4AII protein levels increased substantially (∼8 fold) over the 5 day differentiation period (Figs. 1B, C). PDCD4 levels, a negative regulator of eIF4A capable of sequestering eIF4A and preventing its entry into the eIF4F complex, were not altered during the differentiation process (Fig. 1B). As expected, expression of myogenin, a muscle specific transcription factor, was induced upon differentiation of C2C12 cells (Fig. 1B). Moreover, we observed an increase in protein synthesis rates throughout the differentiation process. (Fig 1D). To determine if the changes in eIF4AII levels during C2C12 differentiation was accompanied by an increase in eIF4AII_c_ assembled into the eIF4F complex, we isolated eIF4F from C2C12 cells during the first 3 days of exposure to DM. In a manner that paralleled eIF4A_f_ levels (Fig. 1B), eIF4AI_c_ levels were relatively constant over the first three days of exposure to DM (Fig. 1E). In contrast, an increase in eIF4AII_c_ occurred over the course of differentiation (Fig. 1E, lanes 9–12). This correlated with an increase in eIF4E and eIF4G during the first day of differentiation (Fig. 1E, compare lanes 2 and 3 to 1) suggesting the formation of new eIF4F complexes.

**Figure 2:**
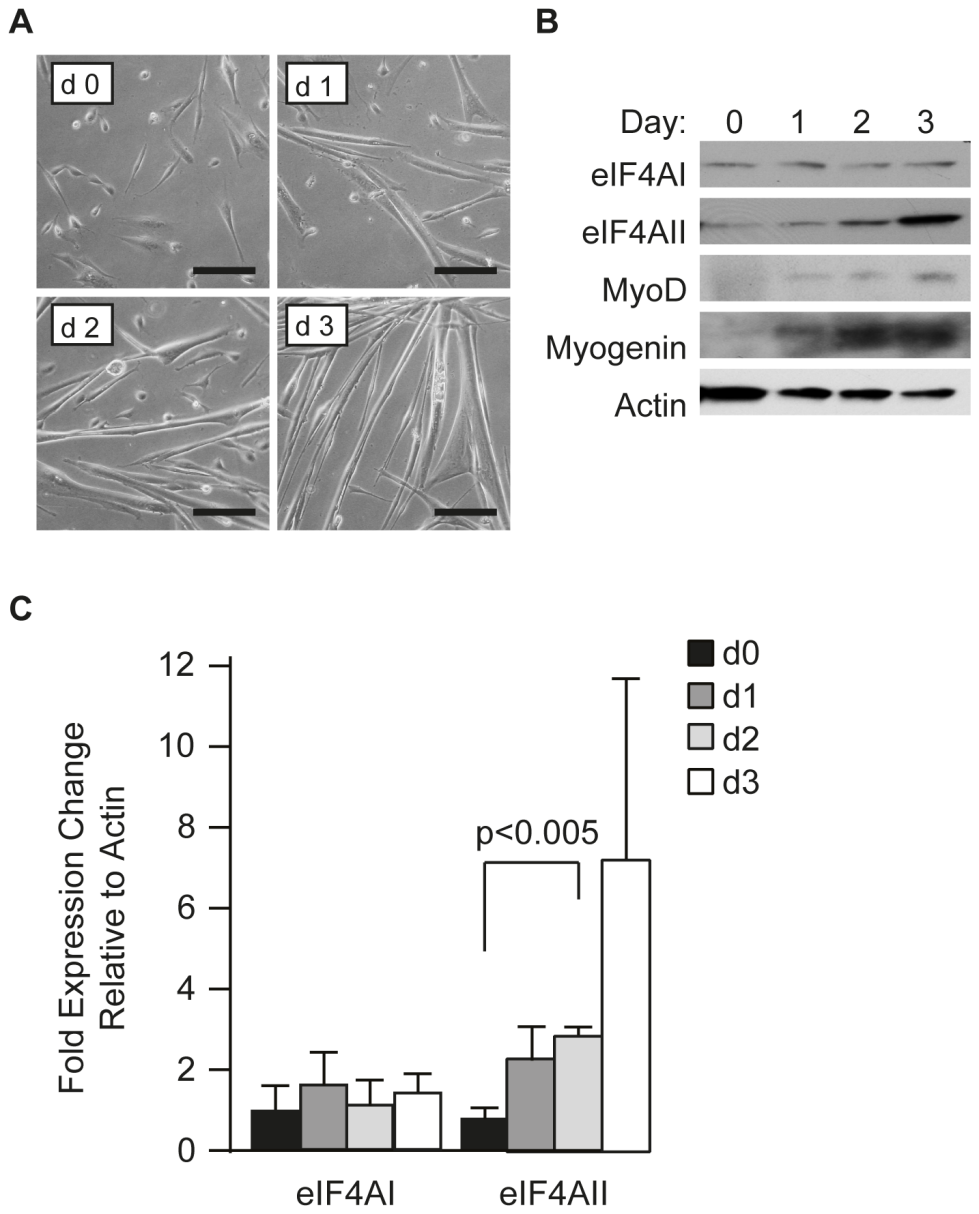
Expression of eIF4AI and eIF4AII during primary myoblast differentiation. (**A**) Phase contrast images of primary myoblasts induced for differentiation over the indicated number of days (d). Scale bars represent 50 µm. (**B**) Western Blot analysis documenting expression levels of the indicated proteins during primary myoblast differentiation. (**C**) Quantification of changes in eIF4AI and eIF4AII protein levels relative to β-actin. n = 3±SEM.

We repeated our analysis of eIF4AI and eIF4AII levels during differentiation of primary myoblast cultures. Differentiation of primary myoblasts occurs much earlier than observed with C2C12 cells with the appearance of myotubes occurring two days after induction of differentiation (Fig 2A). During this process, eIF4AI expression remained unchanged while eIF4AII levels again increased (Fig. 2B, C). These results indicate that increased eIF4AII levels are a feature of muscle cell differentiation.

### MyoD regulates eIF4AII during muscle cell differentiation

**Figure 3:**
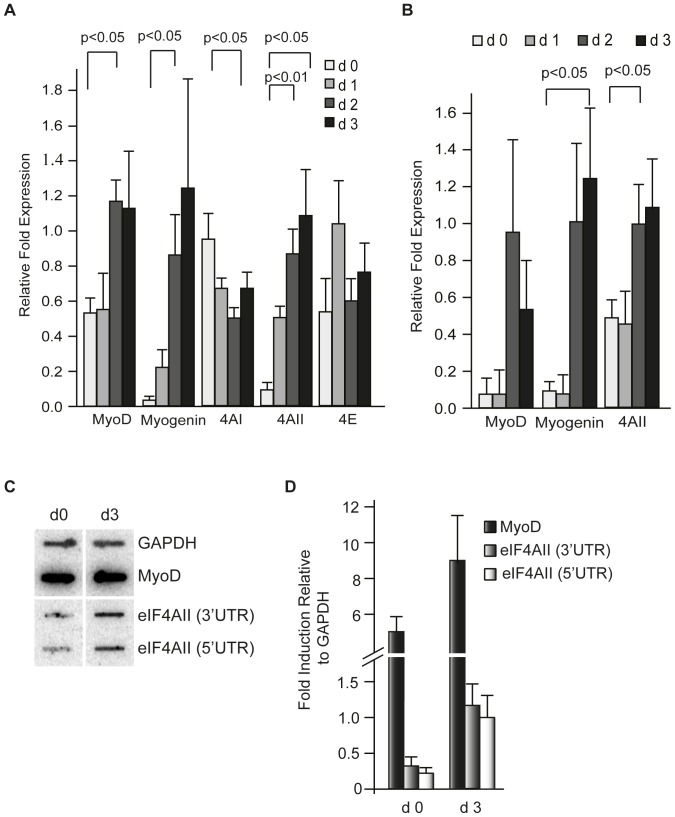
Transcriptional changes in eIF4AII mRNA levels during C2C12 differentiation. (**A**) Changes in eIF4AI and eIF4AII mRNA levels during C2C12 cell differentiation. mRNA levels were determined by RT-qPCR and are standardized to GAPDH levels. n = 3±SEM. (B) Transcriptional changes in eIF4AI and eIF4AII mRNA levels during primary myoblast differentiation. mRNA levels were determined by RT-qPCR and are standardized to GAPDH levels. n = 4±SEM. (**C**) Nuclear Run-On analysis of GAPDH, MyoD and eIF4AII transcription in C2C12 cells at days 0 and 3 after induction of differentiation. Probes targeting the 5′ and 3′ UTRs of eIF4AII were used to distinguish the transcript from that of eIF4AI. (**D**) Quantiation of nuclear run-on experiments. Changes in eIF4AII transcription was quantified using a Typhoon Scanner (GE Healthcare) (values are normalized to GAPDH mRNA levels which did not change over this time period).

To determine if the changes in eIF4AII protein levels observed during myogenesis correlated with an increase in eIF4AII mRNA levels, we analyzed RNA samples isolated at different points post-induction of differentiation by RT-qPCR (days 0 to 3) (Fig. 3A). The levels of MyoD and myogenin mRNA increased ∼2 and 40-fold respectively during this time period - consistent with induction of muscle cell differentiation. Levels of eIF4E mRNA however, were not significantly altered during this time period and eIF4AI levels even slightly decreased. In contrast, there was a significant change in eIF4AII mRNA levels, with an increase of ∼10-fold occurring over the first 3 days after induction of differentiation (Fig. 3A). Analysis of RNA levels from differentiating primary myoblasts revealed increases in MyoD and myogenin mRNA as expected. A modest, but significant, increase in eIF4AII mRNA levels was also noted (∼2 fold increase two days after induction of differentiation) (Fig. 3B).

To determine if the increase in eIF4AII levels was due to a transcriptional response, we performed nuclear run-on experiments. Using probes located within the 5′ or 3′ untranslated regions (UTRs) to distinguish between eIF4AII from eIF4AI, we found that transcription of the *eIF4AII gene* increased 3–4 fold following induction of differentiation (Figs. 3C, D). Taken together, these results indicate that eIF4AII mRNA levels are transcriptionally induced upon muscle cell differentiation.

**Figure 4:**
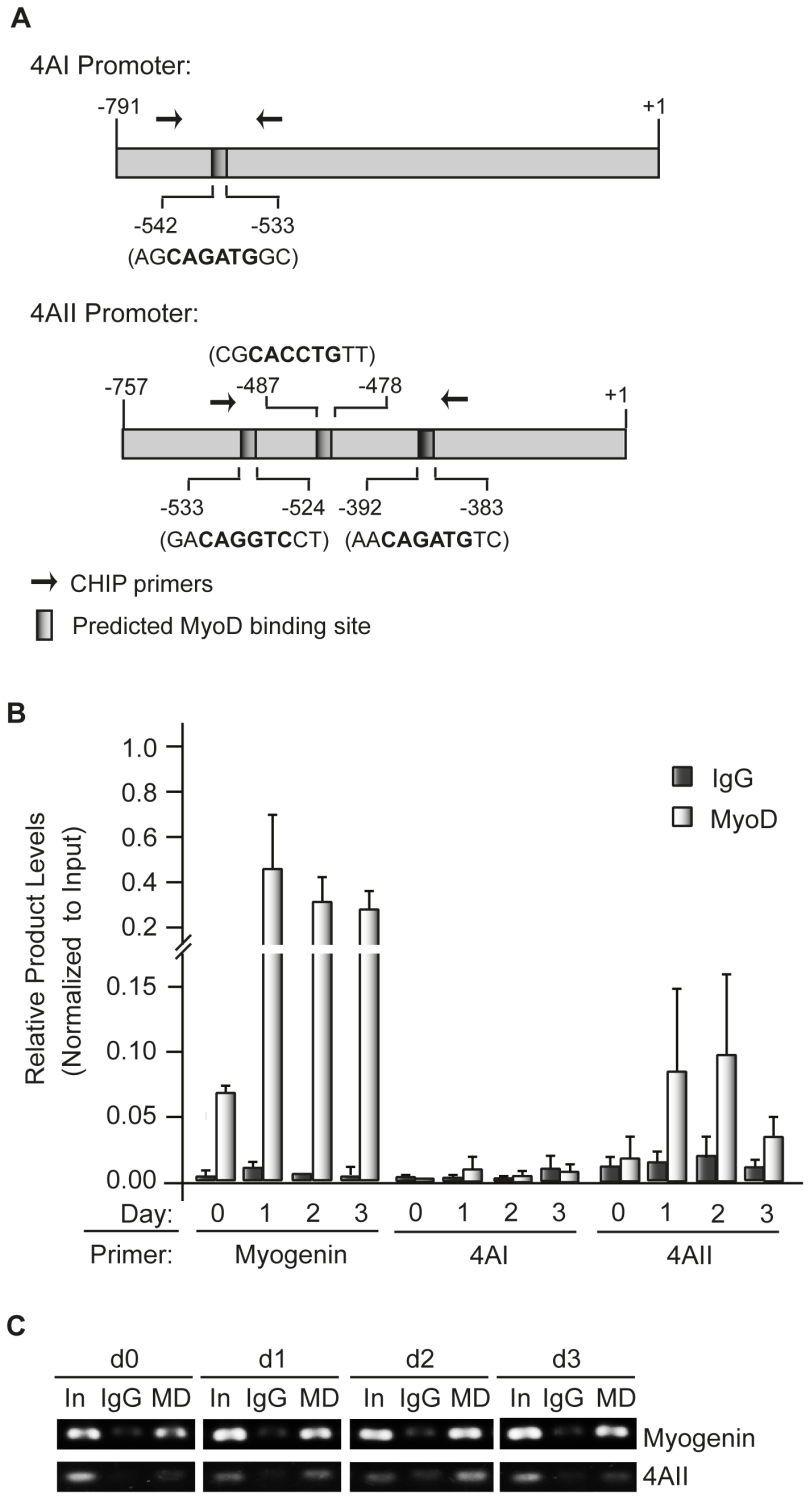
MyoD binds to the endogenous eIF4AII promoter following induction of C2C12 differentiation. (**A**) Schematic representation of the eIF4A and eIF4AII promoters showing the relative position and nucleotide sequence of putative MyoD binding sites. Positions are relative to the transcription start site (+1). Arrows denote the relative position of the primers used in the ChIP assay. (**B**) ChIP assays performed with C2C12 extracts prepared on the indicated days following induction of differentiation. Equivalent amounts of crosslinked chromatin were immunoprecipitated using either an anti-MyoD antibody or an IgG control. The presence of eIF4AI and eIF4AII promoter sequences in the immunoprecipitations was evaluated by qPCR. Primers to the myogenin promoter were used as control and values are normalized to input levels. The input sample represents 5% of the initial DNA material following sonication before immunoprecipitation. n = 4±SEM. (**C**) Products of qPCRs from ChIP assays performed in (B) (In-Input, Ig- IgG elution, MD- MyoD elution).

The muscle differentiation program is driven by the MyoD master regulator [15]. The binding of MyoD to elements in the promoter region of its target genes, such as myogenin, triggers the myogenic differentiation program. Given the increase in eIF4AII mRNA levels following exposure of cells to DM we wished to assess whether eIF4AII was a MyoD target. Examination of the 5′ proximal *eIF4AI* and *eIF4AII* promoters revealed the presence of one and three putative MyoD binding sites, respectively (Fig 4A). To directly assess if MyoD was present on either *eIF4AI* or *eIF4AII* promoters upon activation of differentiation, we performed Chromatin Immunoprecipitation (ChIP) assays. As expected, MyoD binding to the myogenin promoter significantly increased one day following induction of differentiation and slowly declined over the next two days (d2–d3) (Figs. 4B, C). No significant binding of MyoD to the *eIF4AI* promoter was detected throughout this period (Figs. 4B). MyoD was clearly detected at the *eIF4AII* promoter 1–2 days following induction of differentiation (Figs. 4B, C).

**Figure 5:**
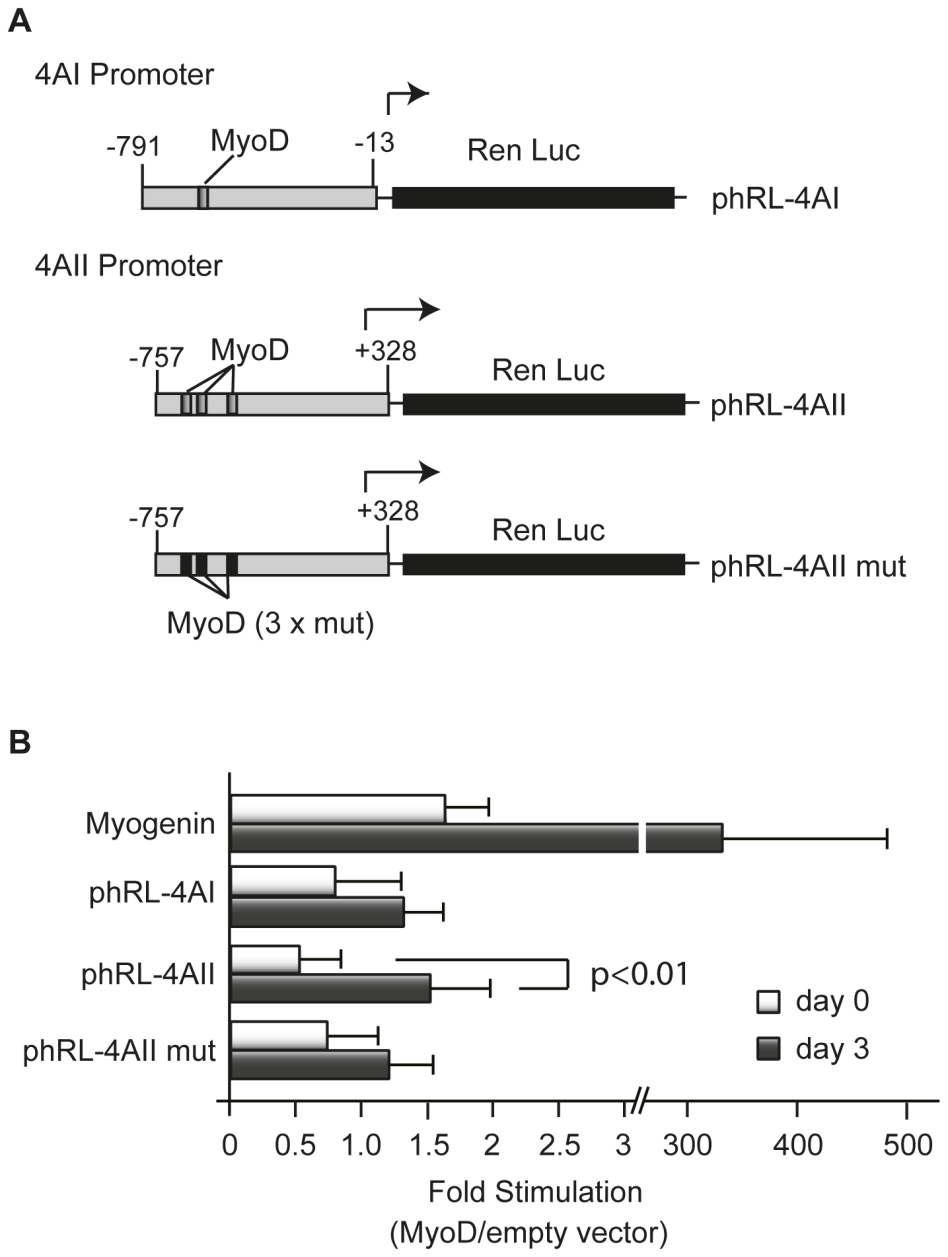
The proximal eIF4AII promoter is activated by MyoD. (**A**) Schematic representation of Renilla Luciferase reporters linked to eIF4AI and eIF4AII proximal promoter sequences. The relative position of putative MyoD binding sites is indicated. (**B**) Transactivation assays involving eIF4AI and eIF4AII reporter constructs and MyoD expression vectors. NIH-3T3 cells were transfected as indicated in the Materials and Methods and extracts prepared on the indicated days following differentiation. Relative light units (RLU) were standardized to protein levels. The ratio of MyoD dependent expression relative to empty expression vector is plotted. n = 4±SEM.

MyoD is able to bind to the promoter of a large number of genes however not all of them are activated by this transcription factor and some are only transiently activated [24], [25]. To determine the contribution of the predicted MyoD binding sites to the eIF4AII transcriptional response, we generated chimeric reporter constructs in which the *eIF4AI* and *eIF4AII* murine promoters were positioned upstream of a *Renilla* luciferase reporter. We then proceeded to mutate all three putative MyoD sites in the *eIF4AII* promoter (Fig. 5A). Co-transfection assays with a MyoD expression vector was performed in NIH 3T3 cells, rather than C2C12 cells to distinguish a MyoD-based response from a C2C12 differentiation program-dependent response [23]. Co-expression of MyoD and a myogenin promoter reporter resulted in robust (200-fold) induction of luciferase activity (Fig. 5B). MyoD exerted a modest, but significant, transcriptional response on the eIF4AII reporter construct (∼3-fold increase), which was not apparent with phRL-4AI reporter (Fig. 5B). Abolishing the MyoD binding sites in phRL-4AII blunted the MyoD induced transcriptional response consistent with the aforementioned MyoD effect on phRL-4AII expression being mediated through these sites.

### Co-suppression of eIF4AI and eIF4AII impairs muscle differentiation

**Figure 6:**
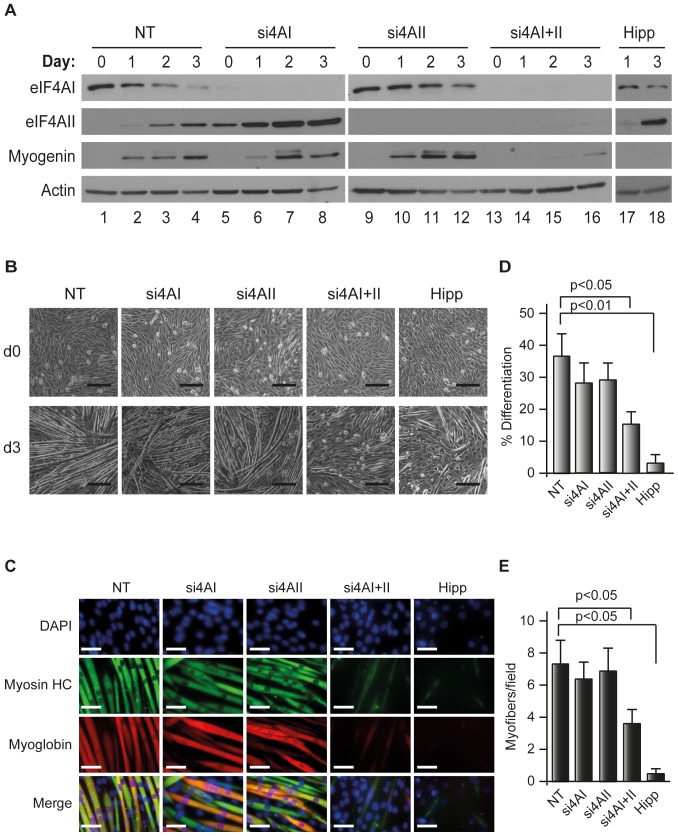
C2C12 differentiation is curtailed upon suppression of eIF4AI and eIF4AII. (**A**) Western blot analysis of siRNA- or hippuristanol- treated C2C12 cells at the indicated times following induction of differentiation. Blots were probed with antibodies to the proteins indicated to the left of the panels. (**B**) Phase contrast images of siRNA- or hippuristanol- (125 nM) treated C2C12 myoblasts at initiation (Day 0) or three days post-induction of differentiation. Scale bars represent 50 µm. (**C**) Immunofluorescence images of siRNAs or hippuristanol treated C2C12 cells at day 3 after induction of differentiation. Myofiber formation was analyzed by immunofluorescence using anti-myosin HC and anti-myoglobin antibodies. Scale bars represent 50 µm. (**D**) Fusion index of muscle fibers (% Differentiation). n = 3±SEM. (**E**) Quantification of myofibers/field. n = 3±SEM.

We next investigated whether the myogenic program was sensitive to fluctuations in eIF4AI or eIF4AII levels. Using RNAi, we transiently suppressed expression of the individual eIF4A isoforms in differentiating C2C12 cells and evaluated the consequences on myogenesis (Fig. 6A, B). As assessed morphologically by phase contrast images and by immunoflourescent staining for myosin heavy chain (HC) and myoglobin, reductions in eIF4AI levels were insufficient to block differentiation (Fig. 6B, C). As well, preventing induction of eIF4AII levels during the myogenic process (Fig. 6A, lanes 9–12) had little notable effect on C2C12 differentiation (Figs. 6B–E). In contrast, suppression of both eIF4AI and eIF4AII by RNAi or the small molecule inhibitor, hippuristanol [9], [12], profoundly blocked myogenesis (Figs. 6B, C). Taken together our results indicate that the myogenic program can tolerate large fluctuations in individual eIF4AI or eIF4AII levels, but not when both isoforms are suppressed or their function inhibited.

## Discussion

A previous analysis of eIF4A levels in differentiating C2C12 muscle cells reported little differences during the first 3 days of differentiation [17] in contrast to our findings (Fig. 1B). However, in that study the levels of individual eIF4A isoforms were not assessed, but rather total eIF4A levels were probed. As well, we used a different induction protocol to activate the myogenic program that avoided the use of insulin since this has been linked to hypertrophy in C2C12 muscle fibers as a consequence of sustained Akt/mTOR signaling [26], [27]. The elevated levels of eIF4AII observed during differentiation correlated with an increase in eIF4AII_c_ levels, a finding indicating that eIF4A availability may limit eIF4F complex formation during C2C12 differentiation (Fig. 1E). One mechanism by which eIF4A may be limiting for eIF4F assembly is via its association with PDCD4 - a tumor suppressor gene product whose association with eIF4A is under PI3K/mTOR regulation [28]. Activation of the PI3K/mTOR pathway has been reported to lead to phosphorylation and degradation of PDCD4 in a proteasome-dependent manner with a concomitant increase in eIF4A availability [28]. However, PDCD4 levels do not significantly change during C2C12 differentiation (Fig. 1B) making it unlikely that destabilization of PDCD4 is responsible for the increase in eIF4AII_c_ that we observed upon activation of myogenesis. The increase in eIF4AII levels however coincided with an increase in eIF4AII transcription that was stimulated by MyoD (Figs. 3–5). The transcription of eIF4AII, but not eIF4AI, is also consistent with the temporal activation of other MyoD regulated genes, such as myogenin. The weak transcriptional effect of MyoD in our surrogate eIF4AII promoter assays may reflect the contribution of additional MyoD sites present outside of the proximal promoter area tested or may be due to cell-type specific effects (e.g. binding to E-proteins [24] or different histone acetylation events [25]) since the promoter assays were performed in NIH 3T3 cells. As well, our results do not exclude the possible contribution of additional, post-transcriptional events contributing to increased eIF4AII protein levels.

Most evidence suggests that there is one eIF4A molecule per eIF4F unit [29], [30]. This would indicate that the increase in eIF4AII_c_ observed in the eIF4F complex during differentiation (Fig. 1E, compare lane 12 to 9) is a consequence of eIF4AII forming either new complexes with eIF4E and eIF4G during C2C12 differentiation and/or displacing eIF4AI_c_ from existing eIF4F complexes (Fig. 1E, compare lanes 9 and 12). We do not favour the latter possibility given that eIF4AI_c_ levels did not change during the early stages of myogenesis (Fig. 1E, compare lanes 9–12). The results rather suggest that new eIF4F complexes containing eIF4AII_c_ are formed during initiation of the differentiation program. This would result in higher levels of eIF4F complexes in which case we would expect a relief in mRNA competition for ribosome recruitment consistent with the increase in translational activity observed (Fig. 1D).

We have not investigated the mechanism(s) responsible for the declining eIF4AI levels observed later in the differentiation process (Fig. 1B; d3–d4), but we noted a decrease in eIF4AI mRNA levels over the first 3 days of myogenesis (Fig. 1C). The lag between the decrease in eIF4AI mRNA levels and the lower eIF4AI levels may be a consequence of eIF4AI's relatively long half-life (t_1/2_>24 h) [7].

Meijer et al. [8] has implicated eIF4AII, but not eIF4AI, in let-7-mediated translational repression suggesting that increases in eIF4AII levels should be associated with repression of mRNAs under miRNA regulation. PDCD4 and MyoD have been reported to be under mir21 [31] and miR203b [32] regulation, respectively, yet we did not see decreases in expression of either of these factors upon eIF4AII suppression (Figs. 1B, 2B, 3A, 3B). Hence, this mode of regulation either is not at play during muscle cell differentiation or is restricted to let-7-mediated regulation.

Suppression of the individual eIF4AI or eIF4AII isoforms did not curtail activation or execution of the myogenic program (Fig. 6). This suggests that the myogenic program is “buffered” against short-term fluctuations in eIF4A levels. An effect however was noticed when both isoforms were inhibited by RNAi or hippuristanol - indicated either that reductions below a certain threshold are not tolerated or there exists a synthetic lethal relationship between eIF4AI and eIF4AII (Fig. 6). Although we cannot exclude an effect on the differentiation program due to inhibition of protein synthesis upon co-suppression of eIF4AI and eIF4AII, our previous experiments suggest this may not be the primary or complete mechanism. We have previously shown that suppression of eIF4AII does not inhibit ^35^S-methionine incorporation in HeLa or HEK293 cells, whereas suppression of eIF4AI or co-suppression of both eIF4AI and eIF4AII reduced protein synthesis to the same extent (∼40–50% wild-type levels) [7]. Our failure to observe a response on C2C12 differentiation when only eIF4AI is suppressed suggests that inhibition of global protein synthesis is unlikely the sole reason for the differences on C2C12 differentiation seen between eIF4AI versus eIF4AI/eIF4AII suppression. Recently we have provided data indicating that eIF4AI and eIF4AII may not have completely overlapping functions since eIF4AII was unable to rescue the translational block imposed upon suppression of eIF4AI [7]. Here we document reciprocal regulation of eIF4AI and eIF4AII during C2C12 differentiation. Although it is unclear why such a shift in isoform abundance would be required, it may be that the target spectrum of the eIF4A isoforms during initiation is not equivalent or that the need for elevated eIF4AII levels is required for a role outside of that for ribosome recruitment.

## Conclusions

Our results demonstrate that eIF4A isoforms are differentially regulated during muscle cell differentiation. We report that the eIF4AII isoform is regulated by MyoD during myogenesis, underscoring regulatory differences between eIF4AI and eIF4AII. Furthermore, we demonstrate that the presence of both eIF4A isoforms is necessary for correct execution of the differentiation program.
